# Identification of heel bone mineral density as a risk factor of Alzheimer’s disease by analyzing large-scale genome-wide association studies datasets

**DOI:** 10.3389/fcell.2023.1247067

**Published:** 2023-11-30

**Authors:** Feng Gao, Rongrong Pan, Taixuan Fan, Lingling Liu, Haile Pan

**Affiliations:** ^1^ Department of Orthopedics, The Second Affiliated Hospital of Harbin Medical University, Harbin, China; ^2^ Department of Orthopedics, Huangshan People’s Hospital, Huangshan, China

**Keywords:** Mendelian randomization, Alzheimer’s disease, bone mineral density, genome-wide association studies, inverse-variance weighted meta-analysis

## Abstract

**Introduction:** Both low bone mineral density (BMD) and Alzheimer’s disease (AD) commonly co_occur in the older adult. Until now, the association between AD and BMD has been widely reported by observational studies. However, Mendelian randomization (MR) studies did not support the causal association between BMD and AD. We think that the lack of significant causal association between AD and BMD identified by recent MR studies may be caused by small number of potential instrumental variables.

**Methods:** We conduct a MR study to evaluate the causal effect of heel BMD on the risk of AD using 1,362 genome-wide significant and independent (*p* < 5.00E-08) heel BMD genetic variants as the potential instrumental variables, which are identified by a large-scale genome wide association study (GWAS) of heel BMD in 394,929 UK Biobank individuals. Using these 1,362 genome-wide significant and independent heel BMD genetic variants, we extracted their corresponding AD GWAS summary results in IGAP AD GWAS dataset (*n* = 63,926) and FinnGen AD GWAS dataset (*n* = 377,277). Five methods including inverse-variance weighted meta-analysis (IVW), weighted median, MR-Egger, MR-PRESSO, and MRlap were selected to perform the MR analysis. 951 of these 1,362 genetic variants are available in AD GWAS dataset.

**Results:** We observed statistically significant causal effect of heel BMD on the risk of AD using IVW in IGAP AD GWAS dataset (OR = 1.048, 95%CI: 1.002–1.095, *p* = 0.04) and FinnGen AD GWAS dataset (OR = 1.053, 95% CI:1.011–1.098, *p* = 0.011). Importantly, meta-analysis of IVW estimates from IGAP and FinnGen further supported the causal effect of heel BMD on the risk of AD (OR = 1.051, 95% CI: 1.02–1.083, *p* = 0.0013).

**Discussion:** Collectively, our current MR study supports heel BMD to be a risk factor of AD by analyzing the large-scale heel BMD and AD GWAS datasets. The potential mechanisms underlying the association between heel BMD and AD should be further evaluated in future.

## Introduction

Both low bone mineral density (BMD) and Alzheimer’s disease (AD) commonly co-occur in the older adult. Until now, the association between AD and BMD has been widely reported. The Rotterdam Study in 3,651 participants showed that participants with lower BMD at the femoral neck were associated with increased risk of all-cause dementia with Hazard ratio (HR) = 1.12, 95% confidence interval (CI): 1.02–1.23, and AD with HR = 1.14, 95% CI: 1.02–1.28 during the whole follow-up (Xiao et al., 2023). During the first 10 years follow-up, the lowest tertiles of femoral neck BMD, total body BMD, and trabecular bone score were associated with increased risk of dementia, with HR = 2.03, 95% CI: 1.39–2.96, HR = 1.42, 95% CI: 1.01–2.02, and HR = 1.59, 95% CI: 1.11–2.28, respectively (Xiao et al., 2023). Evidence from 71 early stage AD patients and 69 non-demented older adult controls showed the reduced BMD in early stage AD, which further contributed to brain atrophy and memory decline (Loskutova et al., 2009). Evidence from 150 AD cases and gender and age-matched 150 healthy controls further showed the reduced BMD in AD (Kumar et al., 2021). Evidence suggested significantly increased bone metabolic biomarkers and reduced BMD in early stage AD by analyzing the data from 42 male early stage AD and 40 age-matched healthy older controls (Pu et al., 2020). A community-based prospective cohort study in 987 participants including 610 women and 377 men showed significant relation between low femoral neck BMD and increased risk of AD (relative risk (RR) = 2.37) and all-cause dementia (RR = 2.24) in women but not in men (Tan et al., 2005). The Framingham Offspring Cohort study in 1905 participants further indicated that the increased femoral neck BMD contributed to better performance and lower white matter hyperintensity burden (Stefanidou et al., 2021).

In addition to these observational studies, several Mendelian randomization (MR) studies have also evaluated the bidirectional causal effect between BMD and AD by analyzing multiple large-scale genome wide association study (GWAS) datasets. Xu and others found no significant causal effect of AD on BMD at different sites including femoral neck, forearm, heel, lumbar spine, and total body, and different ages including 0–15, 15–30, 30–45, 45–60, and over 60 years (Hu et al., 2023). Meanwhile, Xu and others identified no evidence of causal effect of BMD at different sites (femoral neck, forearm, heel, lumbar spine, and total body) and different ages (0–15, 15–30, 30–45, 45–60, and over 60 years) on the risk of AD (Hu et al., 2023). MR findings supported the lack of significant causal effects of AD on BMD at different sites including femoral neck, lumbar spine, and forearm (Cui et al., 2020; Tang et al., 2023). However, Nethander and others found a significant causal effect of AD on hip fracture (OR = 1.07, 95% CI: 1.05–1.10, and *p* = 1.9 × 10^−12^, although lack of significant genetic correlation between AD and hip fracture (Nethander et al., 2022).

We think that the lack of significant causal association between AD and BMD identified by recent MR studies may be caused by small number of genetic variants as the potential instrumental variables. For example, Xu and others selected 68, 3, 16, 19 and 296 genetic variants as potential instrumental variables for total body, forearm, femoral neck, lumbar spine, and heel BMD, respectively (Hu et al., 2023). Meanwhile, Xu and others selected 7, 1, 9, 18 and 18 genetic variants as the potential instrumental variables for total body BMD at different ages including 0–15, 15–30, 30–45, 45–60, and over 60 years (Hu et al., 2023). Here, we conduct an updated MR study to evaluate the causal effect of heel BMD on the risk of AD using 1,362 genome-wide significant and independent heel BMD genetic variants (Kim, 2018).

## Materials and methods

### Mendelian randomization design

**FIGURE 1:**
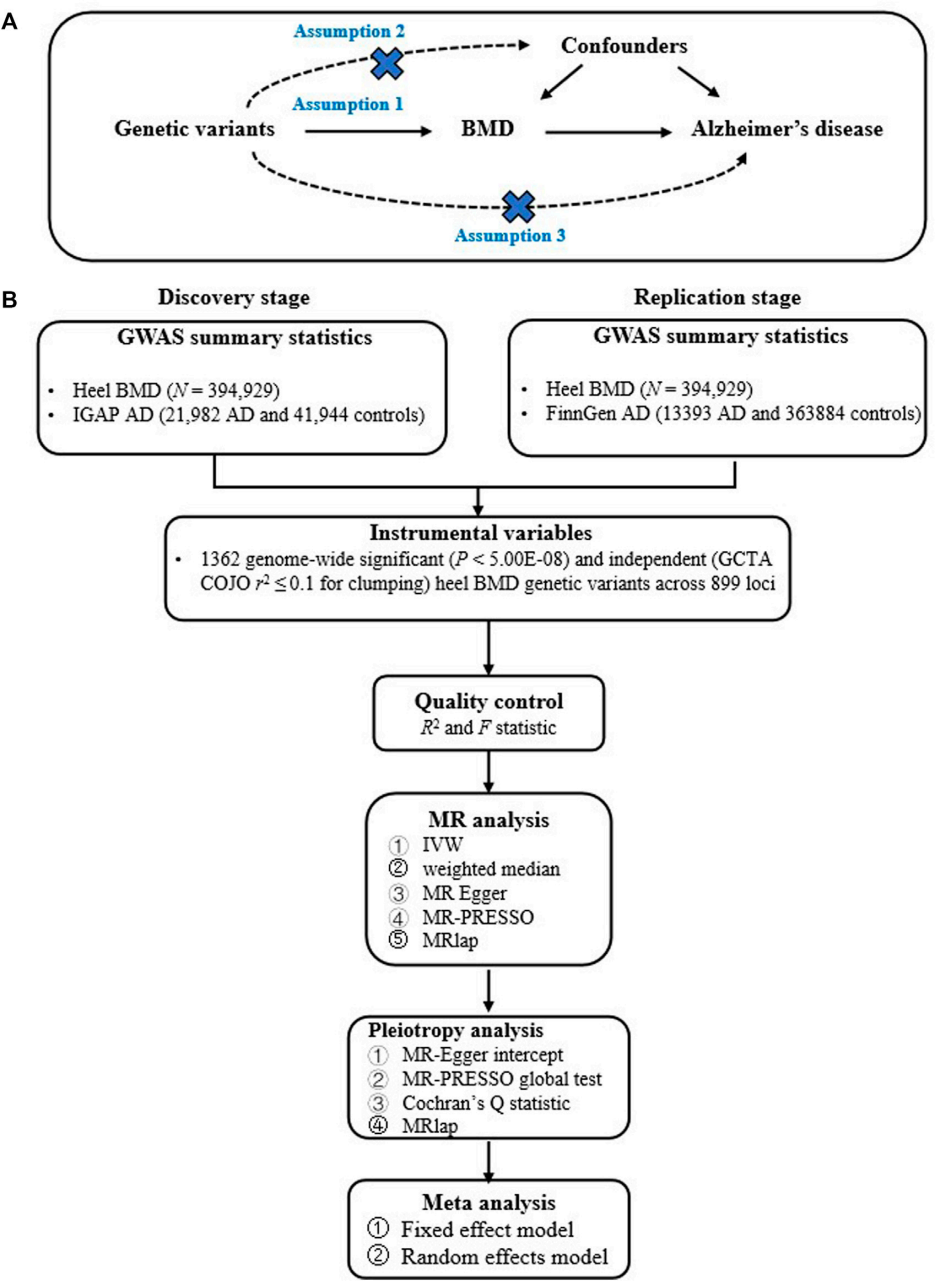
Provides the flow chart of our current study design. **(A)** provides the instrumental variable assumptions; **(B)** provides the main analysis flow.

Our current study design is based on a two-sample MR, which estimates the causal effect of an exposure (such as BMD) on an outcome (AD) only using genome-wide significant genetic variants and large-scale GWAS summary statistics. Importantly, two-sample MR must meet the instrumental variable assumptions to ensure the valid causal inference (Davies et al., 2018; de Leeuw et al., 2022; Wootton et al., 2022). First, relevance assumption: genetic variants must be significantly associated with BMD. Second, independence assumption: genetic variants must not be associated with confounders of the BMD-AD. Third, exclusion-restriction assumption: genetic variants must only be associated with the AD via BMD (Davies et al., 2018; de Leeuw et al., 2022; Wootton et al., 2022). Figure 1 provides the flow chart of our current study design.

### Heel BMD genetic variants

We selected 1,362 genome-wide significant (*p* < 5.00E-08) and independent (GCTA COJO *r* ^2^ ≤ 0.1 for clumping) heel BMD genetic variants across 899 loci as the potential instrumental variables, which were identified by a large-scale GWAS of heel BMD (Kim, 2018). This GWAS is performed in UK Biobank participants of European ancestry (Kim, 2018). A total of 394,929 UK Biobank individuals were available for the final GWAS analysis with both genotype and phenotype data (Kim, 2018). Supplementary Table S1 provides the summary statistics for these 1,362 genome-wide significant and independent heel BMD genetic variants. The original study provides more detailed information about these 1,362 genetic variants (Kim, 2018).

### AD GWAS dataset from IGAP

In the discovery stage, we selected a large-scale AD GWAS dataset from the International Genomics of Alzheimer’s Project (IGAP) consisting of 21,982 autopsy-confirmed or clinically confirmed late-onset AD and 41,944 cognitively normal controls of European descent (Kunkle et al., 2019). The IGAP AD GWAS dataset is a meta-analysis of four available AD GWAS datasets. The fist AD GWAS dataset is from Alzheimer Disease Genetics Consortium (ADGC) including 14,428 autopsy-confirmed or clinically confirmed late-onset AD and 14,562 cognitively normal controls. The second AD GWAS dataset is from Cohorts for Heart and Aging Research in Genomic Epidemiology Consortium (CHARGE) consisting of 2,137 autopsy-confirmed or clinically confirmed late-onset AD and 13,474 cognitively normal controls. The third AD GWAS dataset is from European Alzheimer’s Disease Initiative (EADI), which includes 2,240 autopsy-confirmed or clinically confirmed late-onset AD and 6,631 cognitively normal controls. The fourth AD GWAS dataset is from Genetic and Environmental Risk in AD/Defining Genetic, Polygenic and Environmental Risk for Alzheimer’s Disease Consortium (GERAD/PERADES) including 3,177 autopsy-confirmed or clinically confirmed late-onset AD and 7,277 cognitively normal controls (Kunkle et al., 2019). Supplementary Table S2 provides a demographic description of the datasets in each consortium.

### AD GWAS dataset from FinnGen

In the replication stage, we selected a large-scale AD GWAS dataset from FinnGen (release 9) consisting of 13,393 AD with wide definition and 363,884 controls of European descent (G6_AD_WIDE) (Kurki et al., 2023). FinnGen is a large public-private partnership that aims to collect and analyze genome and national health register data from 500,000 Finnish biobank participants (Kurki et al., 2023). The FinnGen AD GWAS summary statistics were downloaded from https://finngen.gitbook.io/documentation/data-download.

### Mendelian randomization analysis

We conducted a MR analysis using three methods including inverse-variance weighted meta-analysis (IVW) (Bowden et al., 2016), weighted median (Bowden et al., 2016), and MR-Egger (Burgess and Thompson, 2017). IVW is widely used in MR analysis as the main analysis method, and IVW assumes all the selected genetic variants to be valid instrumental variables (Burgess et al., 2020). However, IVW will be biased even if only one of these selected genetic variants is invalid (Burgess et al., 2020). We selected weighted median, MR-Egger, MR-PRESSO, and MRlap as the sensitivity analysis methods (Verbanck et al., 2018; Burgess et al., 2020; Mounier and Kutalik, 2023). Weighted median assumes that the majority of genetic variants (>50%) are valid instrumental variables or the majority of weight (>50%) is from the valid instrumental variables (Burgess and Thompson, 2017). Compared with IVW and weighted median, MR-Egger not only tests the directional pleiotropy, but also provides a causal estimate by correcting for the pleiotropy (Burgess and Thompson, 2017). MR-PRESSO (Mendelian Randomization Pleiotropy RESidual Sum and Outlier) could evaluate (MR-PRESSO global test) and correct for (MR-PRESSO outlier test) horizontal pleiotropy using GWAS summary association statistics (Verbanck et al., 2018). MRlap could take into account the weak instrument bias and winner’s curse, and correct for potential sample overlap and the bias of IVW utilizing GWAS summary association statistics (Mounier and Kutalik, 2023). Meanwhile, we test statistical heterogeneity across the selected instrumental variables using Cochran’s Q statistic (Rucker et al., 2008). The significant causal effect of heel BMD on the risk of AD is provided by odds ratio (OR) as well as its corresponding 95% CI, which corresponds to 1 standard deviation (SD) in heel BMD levels. *p* < 0.05 is defined to be the statistical significant. R Version 4.0.3, R package “MendelianRandomization,” and R package “meta” (version 6.0–0) were used to conduct the MR analysis and meta-analysis (Yavorska and Burgess, 2017).

### *R* ^*2*^ and *F* statistic

*R* ^*2*^ is the proportion of the heel BMD variance that is explained by the instrumental variables, an indicator of power for MR (Burgess and Thompson, 2011; Pierce et al., 2011). *F* reflects the “strength” of an instrumental variable or a set of instrumental variables (Burgess and Thompson, 2011; Pierce et al., 2011). *R* ^*2*^ could be calculated by the formula $R^{2}=\sum_{i=1}^{k}2*MAF*\left(1-MAF\right)*{\beta_{i}}^{2}$ , where $\beta_{i}$ is the effect size for instrumental variable *i*, MAF is the minor allele frequency for instrumental variable *i*, and *k* is the number of the instrumental variables (Locke et al., 2015). *F* is a function of $R^{2}$ , the sample size (*n*) and the number of instrumental variables (*k*), $F=\left(\frac{n-k-1}{k}\right)\left(\frac{R^{2}}{1-R^{2}}\right)$ (Burgess and Thompson, 2011; Pierce et al., 2011).

## Results

### Mendelian randomization in IGAP dataset

**FIGURE 2:**
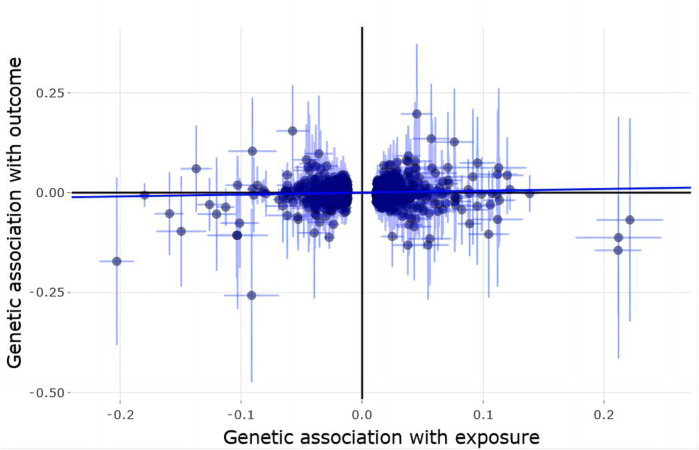
Single estimate about the causal effect of on heel BMD AD in IGAP dataset using IVW. The *x*-axis shows the single effect from each heel BMD genetic variant as the instrumental variable, and standard error, and the *y*-axis shows the single effect from each heel BMD genetic variant as the instrumental variable, and standard error on AD in IGAP. IVW, Inverse-variance weighted.

Using 1,362 genome-wide significant (*p* < 5.00E-08) and independent heel BMD genetic variants, we extracted their corresponding GWAS summary statistics in IGAP AD GWAS dataset. 951 of these 1,362 genetic variants are available in AD GWAS dataset, which are provided in Supplementary Table S3. Using these 951 genetic variants as the potential instrumental variables, we observed statistically significant causal effect of heel BMD on AD risk using IVW method (OR = 1.048, 95% CI: 1.002–1.095, *p* = 0.04). However, weighted median method and MR-Egger method did not indicate any significant association between heel BMD and AD with OR = 0.99, 95% CI: 0.916–1.07, *p* = 0.797 and OR = 0.987, 95% CI: 0.908–1.073, *p* = 0.758, respectively. Table 1 provides the MR results about the association between heel BMD and AD. Figure 2 provides the single causal estimates about the effect of heel BMD on the risk of AD in IGAP dataset using IVW.

**TABLE 1:** MR findings about the association between heel BMD and AD.

Dataset	Method	OR	95% CI	*p*-Value
IGAP	IVW	1.048	1.002–1.095	0.04
IGAP	Weighted median	0.99	0.916–1.07	0.797
IGAP	MR-Egger	0.987	0.908–1.073	0.758
IGAP	MR-PRESSO	1.038	0.994–1.084	0.089
IGAP	MRlap	1.011	0.992–1.032	0.262
FinnGen	IVW	1.053	1.011–1.098	0.011
FinnGen	Weighted median	1.021	0.954–1.094	0.542
FinnGen	MR-Egger	1.044	0.971–1.122	0.239
FinnGen	MR-PRESSO	1.042	1.003–1.084	0.037
FinnGen	MRlap	1.131	0.415–3.079	0.414
Meta-analysis	Fixed effect model IVW	1.051	1.02–1.083	0.0013
Meta-analysis	Random effects model IVW	1.051	1.02–1.083	0.0013
Meta-analysis	Fixed effect model MR-PRESSO	1.041	1.011–1.071	0.0071
Meta-analysis	Random effects model MR-PRESSO	1.041	1.011–1.071	0.0071
Meta-analysis	Fixed effect model MRlap	1.011	0.992–1.032	0.2613
Meta-analysis	Random effects model MRlap	1.011	0.992–1.032	0.2613

IVW, inverse-variance weighted; CI, confidence interval; OR, odds ratio.

There is clear evidence of heterogeneity across these selected instrumental variables with Cochran’s Q statistic = 1,207.3781, degrees of freedom 950, *p* < 1.00E-04, and I^2 = 21.3%. MR-Egger intercept test showed no evidence of pleiotropy with intercept = 0.002, and *p* = 0.094. MR-PRESSO global test showed evidence of horizontal pleiotropy with *p* < 5.00E-04. MR-PRESSO outlier test and MRlap found suggestive and lack of significant association between genetically increased heel BMD and increased risk of AD with OR = 1.038, 95% CI: 0.994–1.084, *p* = 0.089 and OR = 1.011, 95% CI: 0.992–1.032, *p* = 0.262, respectively. Interestingly, MR estimates from MR-PRESSO outlier and MRlap are consistent in direction.

### Mendelian randomization in FinnGen dataset

**FIGURE 3:**
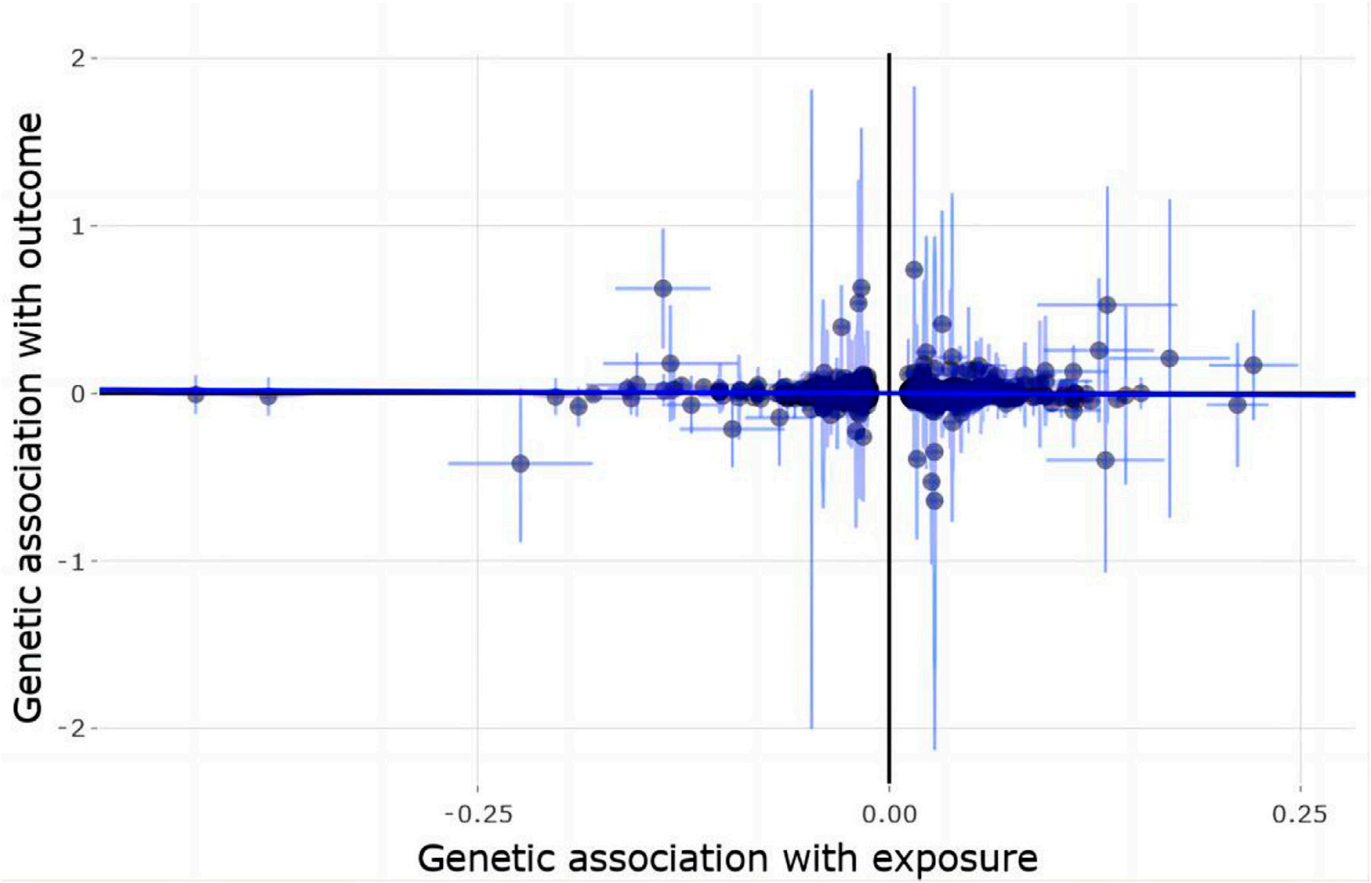
Single estimate about the causal effect of on heel BMD AD in FinnGen dataset using IVW. The *x*-axis shows the single effect from each heel BMD genetic variant as the instrumental variable, and standard error, and the *y*-axis shows the single effect from each heel BMD genetic variant as the instrumental variable, and standard error on AD in FinnGen. IVW, Inverse-variance weighted.

Using 1,362 genome-wide significant (*p* < 5.00E-08) and independent heel BMD genetic variants, we extracted their corresponding GWAS summary statistics in FinnGen AD GWAS dataset. 1,037 of these 1,362 genetic variants are available in FinnGen AD GWAS dataset, which are provided in Supplementary Table S4. Using these 1,103 genetic variants as the potential instrumental variables, we still observed statistically significant causal effect of heel BMD on AD risk using IVW method (OR = 1.053, 95% CI: 1.011–1.098, *p* = 0.011). Interestingly, both weighted median method (OR = 0.99, 95% CI: 0.916–1.07, *p* = 0.797) and MR-Egger method (OR = 0.99, 95% CI: 0.916–1.07, *p* = 0.797) suggested the same directions about the causal effect of heel BMD on AD risk, although lack of statistical significance. Table 1 provides the MR results about the association between heel BMD and AD. Figure 3 provides the single causal estimates about the effect of heel BMD on the risk of AD in FinnGen dataset using IVW.

There is clear evidence of heterogeneity across these selected instrumental variables with Cochran’s Q statistic = 1,338.1074, degrees of freedom 1,036, *p* < 1.00E-04, and I^2 = 22.6%. MR-Egger intercept test showed no evidence of pleiotropy with intercept = 0, and *p* = 0.761. MR-PRESSO global test showed evidence of horizontal pleiotropy with *p* < 5.00E-04. MR-PRESSO outlier test and MRlap found significant and lack of significant association between genetically increased heel BMD and increased risk of AD with OR = 1.042, 95% CI: 1.003–1.084, *p* = 0.037 and OR = 1.131, 95% CI: 0.415–3.079, *p* = 0.414, respectively. Interestingly, MR estimates from MR-PRESSO outlier and MRlap are also consistent in direction.

### Meta-analysis of mendelian randomization in IGAP and FinnGen datasets

Collectively, IVW, MR-PRESSO, and MRlap identified consistent findings in direction about the causal effect of heel BMD on AD risk. We further performed a meta-analysis of IVW, MR-PRESSO, and MRlap estimates from IGAP and FinnGen datasets. Interestingly, meta-analysis of IVW estimates further supported the causal effect of genetically increased heel BMD on the risk of AD using both fixed effect model (OR = 1.051, 95% CI: 1.02–1.083, *p* = 0.0013) and random effects model (OR = 1.051, 95% CI: 1.02–1.083, *p* = 0.0013). Meanwhile, MR estimates from the meta-analysis of MR-PRESSO and MRlap estimates are consistent with MR estimate from the meta-analysis of IVW estimates, as provided in Table 1. Importantly, there is no evidence of heterogeneity across the IVW estimates (Q = 0.03, *p* = 0.8725, and I^2 = 0.0%), MR-PRESSO estimates (Q = 0.02, *p* = 0.8953, and I^2 = 0.0%), and MRlap estimate (Q = 0.05, *p* = 0.8270, and I^2 = 0.0%).

### *R* ^*2*^ and *F* statistic

The sample size for heel BMD GWAS dataset is 394,929 (Kim, 2018). *R* ^2^, the proportion of the heel BMD variance explained by these 1,362 instrumental variables, is 44.74%, as provided in Supplementary Table S1. *F* is 233.998, which shows that there is a strong correlation between these 1,362 instrumental variables and heel BMD, and avoids the potential bias from weak instruments in MR studies.

## Discussion

In this current study, we performed an updated MR analysis to determine the causal effect of heel BMD on the risk of AD by selecting 1,362 genome-wide significant (*p* < 5.00E-08) and independent heel BMD genetic variants as the potential instrumental variables (*n* = 394,929), and extracted the corresponding AD GWAS summary statistics in IGAP AD GWAS dataset (*n* = 63,926) and FinnGen AD GWAS dataset (*n* = 377,277). We found a statistically significant causal effect between increased heel BMD and increased risk of AD in both IGAP (OR = 1.048, 95% CI: 1.002–1.095, *p* = 0.04) and FinnGen (OR = 1.053, 95% CI: 1.011–1.098, *p* = 0.011). Importantly, meta-analysis of IVW estimates from IGAP and FinnGen further supported the causal effect of heel BMD on the risk of AD (OR = 1.051, 95% CI: 1.02–1.083, *p* = 0.0013).

It is known that calcium and vitamin D associates with BMD/fracture, although lack of sufficient clinical evidence to support the clear benefits and harms of alone or combined vitamin D and calcium supplementation to prevent the fractures in community-dwelling adults (Tai et al., 2015; Zhao et al., 2017; Grossman et al., 2018; Jin, 2018). In addition to BMD/fracture, MR studies have also evaluated the casual effect of calcium and vitamin D on the risk of AD. He and others selected 8 genome-wide significant and independent serum calcium genetic variants as the instrumental variables, and found that per 1 standard deviation increase in serum calcium level (about 0.5 mg/dL) contribute to a reduced risk of AD using IVW (OR = 0.57) (He et al., 2020). Shi and others further conducted an updated MR analysis in both discovery stage and replication stage (Shi et al., 2021). In the discovery stage, 14 independent but not genome-wide significant serum calcium genetic variants were selected as the instrumental variables (Shi et al., 2021). In the replication stage, 166 genome-wide significant and independent serum calcium genetic variants were selected as the instrumental variables (Shi et al., 2021). They identified the reduced trend of risk of AD as the serum calcium level increased (Shi et al., 2021). Meanwhile, several studies found reduced risk of AD as the 25OHD level increased (Larsson et al., 2017; Larsson et al., 2018; Wang et al., 2020).

In addition to AD, recent MR studies have also investigated the causal association between BMD and other neurological disease. Cui and others evaluated the causal effects of schizophrenia and bipolar disorder on BMD at different sites including femoral neck, lumbar spine, and forearm (Cui et al., 2020). However, they identified lack of significant causal effect of schizophrenia and bipolar disorder on BMD (Cui et al., 2020). Tang and others found no significant causal effect of schizophrenia, depression, Parkinson’s disease, and epilepsy on BMD at different sites including heel, forearm, lumbar spine, femoral neck, and total body, as well as fractures including leg fracture, arm fracture, heel fracture, spine fracture and osteoporotic fracture (Tang et al., 2023). Yao and others aimed to investigate the bidirectional causal association between BMD/fracture and the risk of MS using large-scale BMD, fracture, and MS GWAS datasets (Yao et al., 2022). However, they only highlighted significant association between fracture and reduced risk of MS (*β* = −0.375, *p* = 0.002) (Yao et al., 2022).

Compared with previous MR studies, our current MR analysis may increase the number of BMD genetic variants from 296 to 1,362 (Hu et al., 2023). Using 296 heel BMD genetic variants to be the potential instrumental variables, Xu and others found no statistically significant association between heel BMD and the risk of AD with OR = 0.991, 95% CI: 0.925–1.061, *p* = 0.794 (Hu et al., 2023). These findings show that the increase in the number of potential instrumental variables may contribute to identify more significant causal association.

However, some limitations still exist in our current MR study. The original GWAS identified 1,362 genome-wide significant and independent heel BMD genetic variants (Kim, 2018). However, only 951 and 1,037 genetic variants are available in IGAP and FinnGen AD GWAS dataset, which are provided in Supplementary Tables S3, S4. These missing genetic variants may influence our current MR results. Our current MR findings should be further verified by adding more heel BMD genetic variants as potential instrumental variables. Second, we only evaluated the effect of heel BMD on the risk of AD. It remains unclear about the effect of AD on the heel BMD, which deserves further investigation by additional studies. Third, we only focus on the heel BMD as the largest number of heel BMD genetic variants (*N* = 1,362) (Kim, 2018), compared to femoral neck (*N* = 16), lumbar spine (*N* = 19), total body (*N* = 68) and heel (*N* = 296) (Hu et al., 2023). Fourth, we did not perform the manual analysis to investigate the potential association of these heel BMD genetic variants with confounders for BMD-AD using Phenoscanner V2, a database of human genotype-phenotype associations (Kamat et al., 2019). We think that this kind of manual analysis only evaluate the known pleiotropy such as calcium and vitamin D levels, but not the unknown pleiotropy. Importantly, Phenoscanner V2 limits the largest number of input either 100 SNPs, 10 genes or 10 genomic regions (Kamat et al., 2019). Therefore, we test the pleiotropy using four statistical methods including MR-Egger intercept test, Cochran’s Q statistic, MR-PRESSO global test, and MRlap.

In summary, our current MR study supports heel BMD to be a risk factor of AD using large-scale GWAS datasets. The potential mechanisms underlying this association should be further evaluated in future.

## Data Availability

The original contributions presented in the study are included in the article/Supplementary Material, further inquiries can be directed to the corresponding author.
